# Clinical outcomes comparison of 10 years versus 5 years of adjuvant endocrine therapy in patients with early breast cancer

**DOI:** 10.1186/s12885-018-4878-4

**Published:** 2018-10-12

**Authors:** Li Li, Bingmei Chang, Xiaoyue Jiang, Xueke Fan, Yingrui Li, Teng Li, Shanshan Wu, Jun Zhang, Seyed Kariminia, Qin Li

**Affiliations:** 1 Department of Oncology, Beijing Friendship Hospital, Capital Medical University, Beijing, 100050 China; 2 Department of Biochemistry and Molecular Biology, Basic Medical College, Shanxi Medical University, Taiyuan, 030001 China; 3 Gastroenterology Department, JinCheng People’s Hospital, Shanxi, 048000 China; 4 Biochemistry and Molecular Biology, Basic Medicine College, Shanxi Medical University, Taiyuan, 050001 China; 5 Statistical Center, Beijing Friendship Hospital, Capital Medical University, Beijing, 100050 China; 6 Department of Hematopathology, University of Texas MD Anderson Cancer Center, Houston, 77030 USA; 7 Molecular and Cellular Oncology, MD Anderson Cancer Center, 1515 Holcombe Blvd, Houston, 77030 USA

**Keywords:** Breast cancer, Extended endocrine treatment, Tamoxifen, Aromatase inhibitor, Disease-free survival

## Abstract

**Background:**

Adjuvant endocrine therapy undoubtedly prolongs the time to recurrence for patients with hormone-positive early breast cancer. Extended endocrine therapy to 10 years or longer has been expected to bring a greater clinical advantage. However, the related research conclusions are controversial.

**Methods:**

Tamoxifen (TAM), Aromatase Inhibitor (AI), Exemestane, letrozole (LET) and anastrozole were used as key words in the literature search. After the patients completed 5 years of adjuvant endocrine treatment, they were allocated to continue endocrine treatment for 5 years or receive placebo/observation for 5 years. Disease-free survival (DFS) and overall survival (OS) were the end points. Systematic assessment was performed using Stata 12.0.

**Results:**

Twelve trials including 30,848 cases were involved. The overall analysis demonstrated that extended endocrine therapy to 10 years significantly prolonged DFS compared with 5 years of endocrine therapy [hazard ratio (HR) = 0.84, 95% CI: 0.73–0.97]. Subgroup analysis showed that DFS was significant prolonged with TAM 5y - AI 5y treatment versus TAM 5y treatment and with (AI and/or TAM) 5y - LET 5y treatment versus (AI and/or TAM) 5y treatment [(HR = 0.61, 95% CI: 0.50–0.76) and (HR = 0.81, 95% CI: 0.71–0.93), respectively]. However, no significant difference was found in the DFS with TAM 5y - TAM 5y treatment versus TAM 5y treatment (HR = 0.97, 95% CI: 0.81–1.17). Overall and subgroup analysis did not demonstrate an OS benefit of therapy extended to 10 years. A DFS benefit of extended endocrine therapy to 10 years was verified in the lymph node-positive subgroup, postmenopausal subgroup and ER+ and/or PR+ subgroup (HR = 058, 95% CI: 0.45–0.75; HR = 0.70, 95% CI: 0.58–0.80; HR = 0.80, 95% CI: 0.67–0.96).

**Conclusions:**

An extended 10 years of endocrine treatment yields a DFS benefit for patients with early breast cancer; (AI and/or TAM) 5y - AI 5y treatment is the optimal choice. ER+ and/or PR+, postmenopausal and lymph node-positive patients are the most suitable groups.

**Electronic supplementary material:**

The online version of this article (10.1186/s12885-018-4878-4) contains supplementary material, which is available to authorized users.

## Background

For patients with hormone-positive early breast cancer, adjuvant endocrine therapy undoubtedly prolongs the time to recurrence [1–3]. Moreover, 5 years of adjuvant endocrine treatment has been verified to be more effective than 1–2 years of treatment [3]. However, the recurrence rate of patients receiving tamoxifen (TAM) increases from 15% at 5 years to 33% at 15 years, and cancer mortality increases from 8.3% at 5 years to 26% at 15 years [3]. To control the increased recurrence rate and mortality rate even after receiving 5-year adjuvant endocrine treatment, extended endocrine therapy to 10 years or longer is expected to bring more clinical advantage. However, the research conclusions are controversial.

Both TAM and aromatase inhibitors (AIs) are used as extended adjuvant endocrine regimens. In MA.17, NSABP-B42, MA-17R trials, the prolonged application of AI to 10 years significantly reduced the recurrence risk after 5 years of adjuvant TAM and/or AI treatment [4–6]. However, in the NSABP-B33 trial, prolonged exemestane (EMT) for 5 years did not significantly decrease the recurrence compared with placebo [7]. In the IDEAL (S1–04) trial, prolonged letrozole (LET) for 5 years also did not prolong the disease-free survival (DFS) compared with prolonged LET for 2.5 years [8]. An extended 10 years of adjuvant TAM compared with 5 years TAM showed mixed results. The ATLAS, aTTom, E4181/E5181 trials showed significant recurrence reduction by 10 years of TAM treatment compared with 5 years of TAM treatment; however, the Scottish trial demonstrated no benefit of extended adjuvant TAM [9–12]. Additionally, the National Surgical Adjuvant Breast and Bowel Project (NSABP) B-14 reported extended TAM by more than 5 years led to a shorter DFS. Furthermore, all extended endocrine treatment did not bring an overall survival (OS) benefit [13].

The real benefit of extended adjuvant endocrine is unclear. The objectives of the present study were to compare the clinical outcomes of extended 10 y versus 5 y of adjuvant endocrine therapy in patients with early breast cancer.

## Methods

### Literature search strategy

This comprehensive analysis was performed according to the Preferred Reporting Items for Systematic Reviews [14]. PubMed (1966–2017), Embase (1974–2017), the annual meeting abstracts of the European Society of Medical Oncology and American Society of Clinical Oncology were searched. Only prospective studies were permitted to be included in the assessment. The initial search used the following MeSH terms: “Breast cancer OR Breast cancers OR Breast carcinoma” AND “Adjuvant endocrine OR Extended endocrine treatment OR Extended adjuvant endocrine treatment OR Prolonged endocrine treatment OR Prolonged adjuvant endocrine treatment”. We also used the following MeSH terms: “Breast cancer OR Breast cancers OR Breast carcinoma” AND “Tamoxifen OR Aromatase Inhibitor OR Exemestane OR letrozole OR anastrozole” AND “Clinical Trial”. The PRISMA Checklist is described in Additional file 1: Table S1.

### Inclusion and exclusion criteria of the trials

All the included studies met the following criteria: 1) All trials were prospective, properly randomized controlled and well matched for factors such as age, gender, tumor stage, performance status, clinical stage, treatment regimen, menopausal status, lymph node status, and hormone status. 2) When the same trial was summed up using different time points, only the trial with complete results and the longest follow-up time was included. 3) The primary endpoint was DFS, and the secondary endpoint was OS. 4) After the patients with early breast cancer had completed 5 years of adjuvant endocrine treatment, they were randomly allocated to continue adjuvant endocrine treatment for 5 years or receive placebo for 5 years (or only undergo observation for 5 years).

The exclusion criteria were as follows. 1) All trials concerning neo-adjuvant endocrine treatment were excluded. 2) Ongoing clinical trials without the results of DFS and OS were excluded. 3) Trials involving concomitant interventions, such as adjuvant chemotherapy or radiotherapy, were excluded.

### Data extraction

Three reviewers (Li Li or Bingmei Chang, Xiaoyue Jiang) independently searched the articles. They screened the articles by reading the titles, abstracts or full texts. Any discrepancy was determined by a third reviewer (Shanshan Wu). The hazard ratio (HR) or risk ratio (RR) and 95% confidence interval (CI) of DFS and OS in each trial were extracted. If a trial only provided a Kaplan-Meier curve, the HR and 95% CI were estimated using the Engauge Digitizer V4.1 screenshot tool and a formula proposed by Parmar [15, 16]. Related statistical data were extracted by an expert at the Statistics Center (Shanshan Wu). The following information were also extracted and summarized: journal name, publication year, author’s name, type of clinical trial, follow-up time, previous endocrine treatment regimens, extended endocrine treatment regimens, primary endpoints, second endpoints, lymph node status, estrogen (ER) + and/ or progesterone (PR) status, and menopausal status. The qualities of the enrolled trials were assessed according to the Cochrane Handbook 4.2.6 for Systematic Reviews of Interventions [17].

### Statistical analysis

Systematic assessment was performed using Stata version 12.0 software (Stata Corporation, College Station, Texas, USA). HR/RR and 95% CI were collected to estimate the clinical efficacy of DFS and OS. An HR > 1.0 indicated more recurrence risk or death risk in the extended endocrine treatment group. In each systematic review, Cochrane’s χ^2^ test was used to evaluate the heterogeneity of the included clinical trials. When the *P*-value of heterogeneity was < 0.05 or I^2^ was > 50%, the random-effects model (REM) was used; otherwise, the fixed-effects model (FEM) was used. Begg’s and Egger’s tests were used to evaluate the publication bias of these trials [18, 19].

## Results

### Characteristics of the included trials

**Fig. 1:**
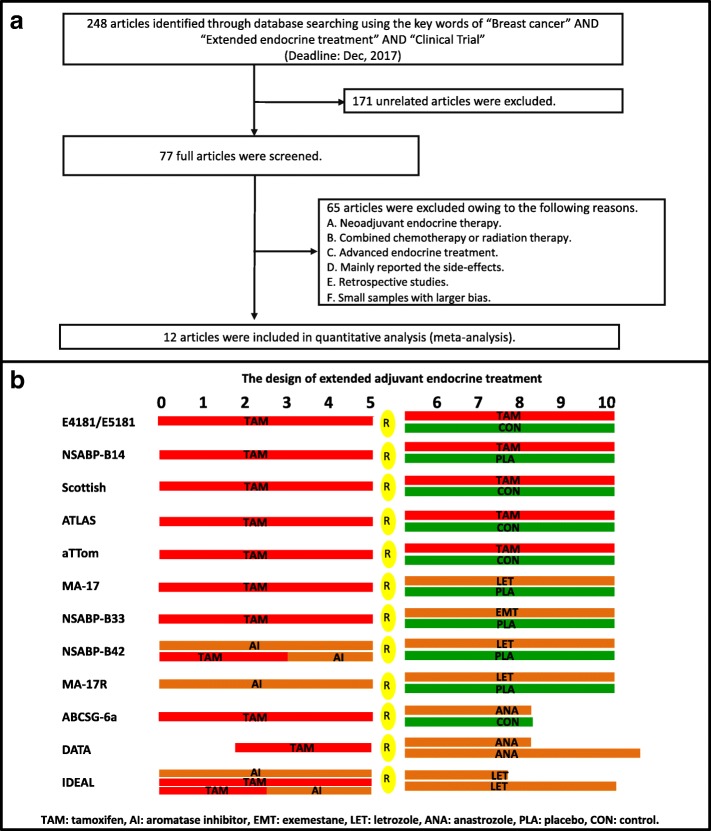
Inclusion of the studies and design of extended endocrine treatments. **a** 12 articles were included in quantitative analysis (meta-analysis), **b** The design of extended adjuvant endocrine treatment

Two hundred forty-eight articles were initially identified through searching the PubMed, Embase and abstracts of International Meeting. One hundred seventy-one articles were excluded by checking the titles and abstracts, and 65 articles were excluded after reading the full text. Finally, 12 trials [4–13, 20] involving 30,848 cases were included in the meta-analysis. The selection flow chart is shown in Fig. 1a, and the design of extended endocrine treatment in all trials is shown in Fig. 1b. The characteristics of the included trials are shown in Table 1. The analysis of Cochrane risk-of-bias showed that the methodological quality of all trials was relatively satisfied and fair.

**Table 1:** Common characteristics of the studies

Trial		Type	Follow up time (y)	Previous treatment	Extended treatment	N	Menopausal State	Lymph Node (+)	ER+ and/ or PR	Primary endpoint
E4181/E5181 (1996)	NR	Full article	5.6	TAM 5y	TAM 5y	100	Premenopausal /Postmenopausal	100/100	73% ER+	DFS
				TAM 5y		93		93/93		
NSABP-B14 (2001)	III	Full article	6.8	TAM 5y	TAM 5y	593	Premenopausal /perimenopausal /Postmenopausal	Negative	ER+	DFS
				TAM 5y	Placebo 5y	579		Negative	ER+	
Scottish trial (2001)	NR	Full article	15.0	TAM 5y	TAM 5y	173	Premenopausal /Postmenopausal	43/90a	66/173b	DFS
				TAM 5y		169		35/89	66/169	
ATLAS (2013)	III	Full article	15.0	TAM 5y	TAM 5y	3428	Premenopausal /perimenopausal /Postmenopausal	1474/3428	ER+	DFS
				TAM 5y		3418		1427/3418	ER+	
aTTom (2013)	III	Abstract	8.6	TAM 5y	TAM 5y	3468	Premenopausal /Postmenopausal	NR	ER+/untested	DFS
				TAM 5y		3485				
MA.17 (2005)	III	Full article	2.5	TAM 5y	LET 5y	2593	postmenopausal	1171/2583	2516/2583	DFS
				TAM 5y	Placebo 5y	2594		1189/2587	2519/2587	
NSABP-B33 (2008)	III	Full article	2.5	TAM 5y	EMT 5y	799	postmenopausal	384/799	775/799	DFS
				TAM 5y	Placebo 5y	799		384/779	759/799	
NSABP-B42 (2016)	III	Abstract	6.9	AI 5y	LET 5y	1959	postmenopausal	NR	1959/1959	DFS
				TAM 3y-AI 2y	Placebo 5y	1964			1964/1964	
MA-17R (2016)	III	Full article	6.3	AI 4.5–6 y	LET 5y	959	postmenopausal	492/959	945/959	DFS
				TAM-AI 4.5–6 y	Placebo 5y	959		494/959	950/959	
ABCSG-6a (2007)	III	Full article	5.2	TAM5y	ANA 3y	387	postmenopausal	132/387	371/387	DFS
				TAM 5y		469		146/469	454/469	
DATA (S1–03) (2016)	III	Abstract	4.1	TAM 2–3y	ANA 6y	931	postmenopausal	561/827	827/827	DFS
				TAM 2–3y	ANA 3y	929		551/827	833/833	
IDEAL(S1–04) (2017)	III	Abstract	6.4	(AI/TAM/TAM -AI) 5y	LET 5y	NR	postmenopausal	partial positive	HR+	DFS
				(AI/TAM/TAM -AI) 5y	LET 2.5y	NR				

*TAM* tamoxifen, *AI* aromatase inhibitor, *EMT* exemestane, *LET* letrozole, *ANA* anastrozole, *ER* estrogen, *HR* hormone receptor, *DFS* disease free survival

### DFS and OS of extended versus routinely adjuvant endocrine treatment

**Fig. 2:**
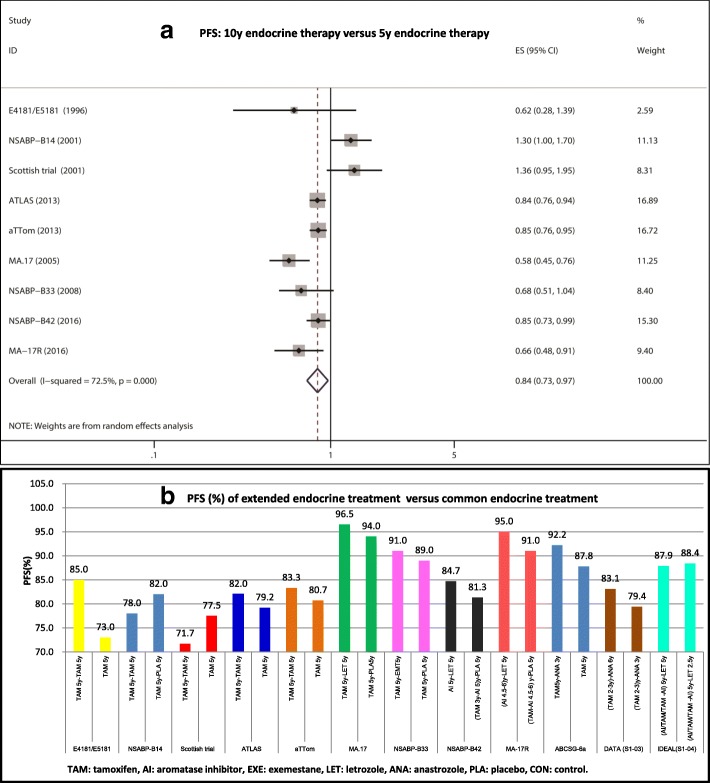
DFS analysis of 10-y endocrine therapy versus 5-y endocrine therapy. **a** PFS: 10y endocrine therapy versus 5y endocrine therapy, **b** PFS (%) of extended endocrine treatment versus common endocrine treatment

As shown in Fig. 2a, twelve trials reported the HR/RR and 95% CIs for DFS and OS. Among them, nine trials compared the prolonged 10 years of endocrine therapy with 5 years of endocrine therapy. Nine trials were divided into three subgroups as follows: subgroup 1 was TAM 5y - TAM 5y versus TAM 5y, subgroup 2 was TAM 5y - AI 5y versus TAM 5y - PLA 5y, subgroup 3 was (AI and/or TAM) 5y - LET 5y versus (AI and/or TAM) 5y - PLA 5y. Three trials compared > 8 y of endocrine therapy with < 8 y of endocrine therapy.

Significant heterogeneity existed among the studies concerning DFS and DFS1; thus, REM was used to analyze the pooled DFS or pooled DFS1 (DFS: I^2^ = 72.5%, *P* = 0.000; DFS1: I^2^ = 74.6%, *P* = 0.003). There was no significant heterogeneity among the studies concerning DFS2 and DFS3; thus, FEM was used to analyze the pooled DFS2 or pooled DFS3 (DFS2: I^2^ = 0.0%, *P* = 0.481; DFS3: I^2^ = 49.0%, *P* = 0.162). The overall analysis demonstrated extended endocrine therapy to 10 years of significantly prolonged DFS compared with 5 years of endocrine therapy (HR = 0.84, 95% CI: 0.73–0.97) (Additional file 2: Figure S1A). Subgroup 2 and subgroup 3 analysis showed that TAM 5y - AI 5y and (AI and/or TAM) 5y - LET 5y treatment significantly prolonged DFS compared with TAM 5y and (AI and/or TAM) 5y treatment, respectively (HR = 0.61, 95% CI: 0.50–0.76; HR = 0.81, 95% CI: 0.71–0.93) (Additional file 2: Figure S1C, 1D). However, no significant difference was found in the DFS between the TAM 5y - TAM 5y group and TAM 5y group (HR = 0.97, 95% CI: 0.81–1.17) (Additional file 2: Figure S1B). The DFS rates of 10 years of endocrine therapy in the NSABP-B14 and Scottish trials were significantly lower than those of 5 years of endocrine treatment, whereas the DFS rates of 10 years of endocrine therapy in seven other trials were increased compared with those of 5 years of endocrine treatment (Fig. 2b).

**Fig. 3:**
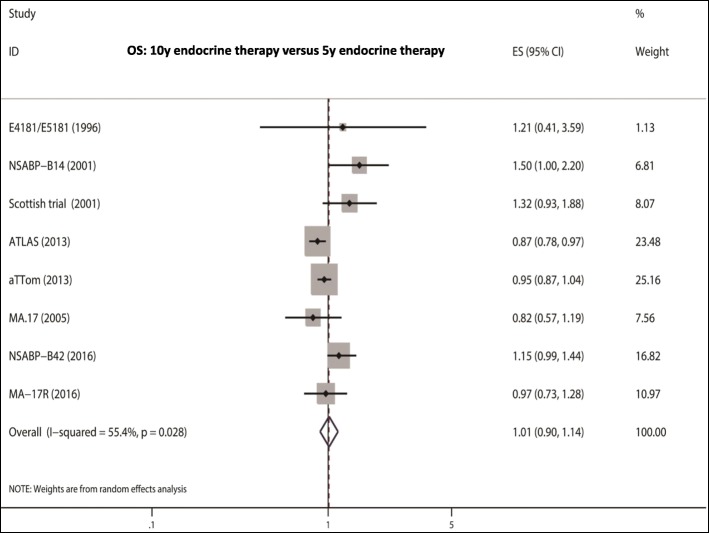
OS analysis of 10-y endocrine therapy versus 5-y endocrine therapy

There was significant heterogeneity among the studies concerning OS and OS1; thus, REM was applied to analyze the pooled OS or pooled OS1 (OS: I^2^ = 55.4%, *P* = 0.028; OS1: I^2^ = 64.2%, *P* = 0.025). No significant heterogeneity was found among the studies concerning OS2 and OS3; thus, FEM was used (OS2: I^2^ = 0.0%, *P* = 0.368; OS3: I^2^ = 0.0%, *P* = 0.323). (HR = 1.01, 95% CI: 0.90–1.14; HR = 1.02, 95% CI: 0.87–1.19; HR = 0.88, 95% CI: 0.64–1.23; HR = 1.09, 95% CI: 0.93–1.28, respectively) (Fig. 3).

As shown in Additional file 3: Figure S2, three trials reported the HR/RR and 95% CIs for DFS and OS, which compared the efficacy of > 8 y of endocrine therapy with that of < 8 y of endocrine therapy. FEM was used to analyze the pooled DFS and pooled OS. Greater than 8 y of endocrine treatment significantly improved DFS compared with < 8 y of endocrine treatment (HR = 0.78, 95% CI: 0.66–0.94). However, no significant improvement was found in OS between the two groups (HR = 0.95, 95% CI: 0.76–1.19).

### DFS analysis in the lymph node-positive subgroup

**Fig. 4:**
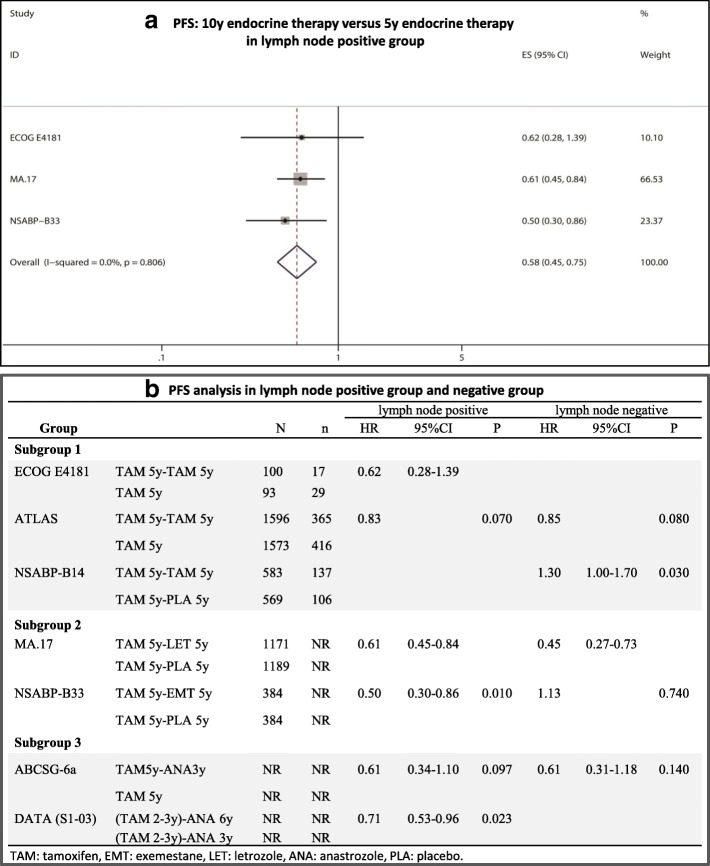
DFS analysis in the lymph node-positive group. **a** PFS: 10y endocrine therapy versus 5y endocrine therapy in lymph node positive group, **b** PFS analysis in lymph node positive group and negative group

As shown in Fig. 4a and b, three trials reported the data of HR/RR and 95% CIs for DFS in the lymph node-positive subgroup. No significant heterogeneity was found among the studies, and FEM was used (I^2^ = 0.00%, *P* = 0.806). The analysis showed that extended endocrine therapy to 10 years significantly improved DFS compared with 5 years of endocrine therapy in the lymph node-positive subgroup (HR = 0.58, 95% CI: 0.45–0.75). In MA.17, positive results of extended endocrine treatment were seen in both the lymph node-positive and -negative groups.

### DFS analysis in the postmenopausal subgroup

**Fig. 5:**
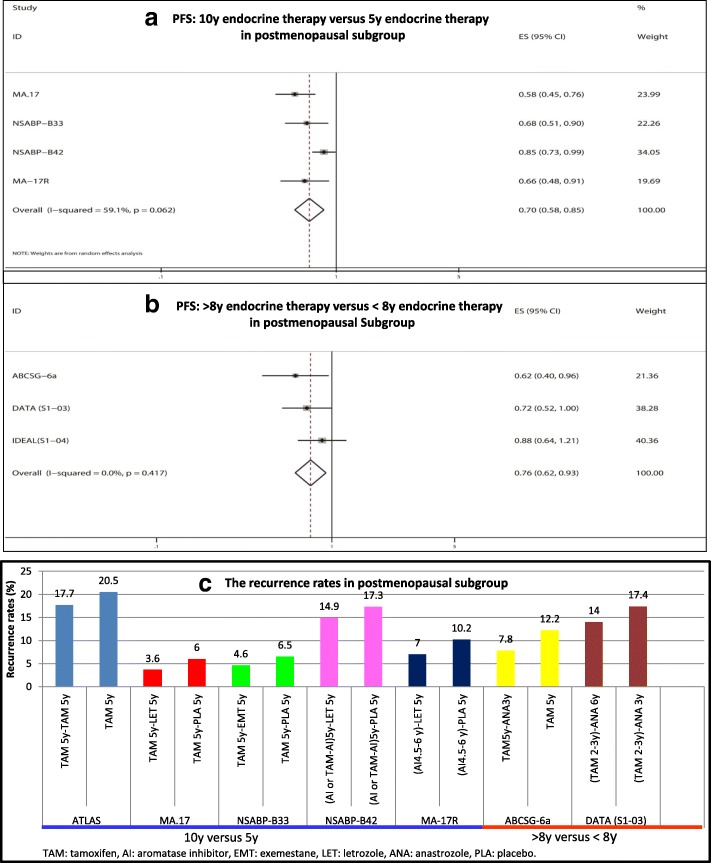
DFS analysis in the postmenopausal subgroup. **a** PFS: 10y endocrine therapy versus 5y endocrine therapy in postmenopausal subgroup, **b** PFS: >8y endocrine therapy versus < 8y endocrine therapy in postmenopausal subgroup

As shown in Fig. 5a and b, seven trials reported the data of HR/RR and 95% CIs for DFS in the postmenopausal subgroup. Postmenopausal subgroup analysis showed that extended endocrine therapy to 10 years significantly improved DFS compared with 5 y of endocrine therapy (HR = 0.70, 95% CI: 0.58–0.85). A similar result was observed between > 8 y of endocrine therapy and < 8 y of endocrine therapy (HR = 0.76, 95% CI: 0.62–0.93). In the ATLAS, MA.17, NSABP-B33, NSABP-B42, MA-17R, ABCSG-6a and DATA trials, the recurrences rates in the extended group and non-extended group were 17.7% versus 20.5%, 3.6% versus 6.0%, 4.6% versus 6.5%, 14.9% versus 17.3%, 7.0% versus 10.2%, 7.8% versus 12.2%, and 14.0% versus 17.4%, respectively.

### DFS analysis in the ER+ subgroup and/or PR+ subgroup

**Fig. 6:**
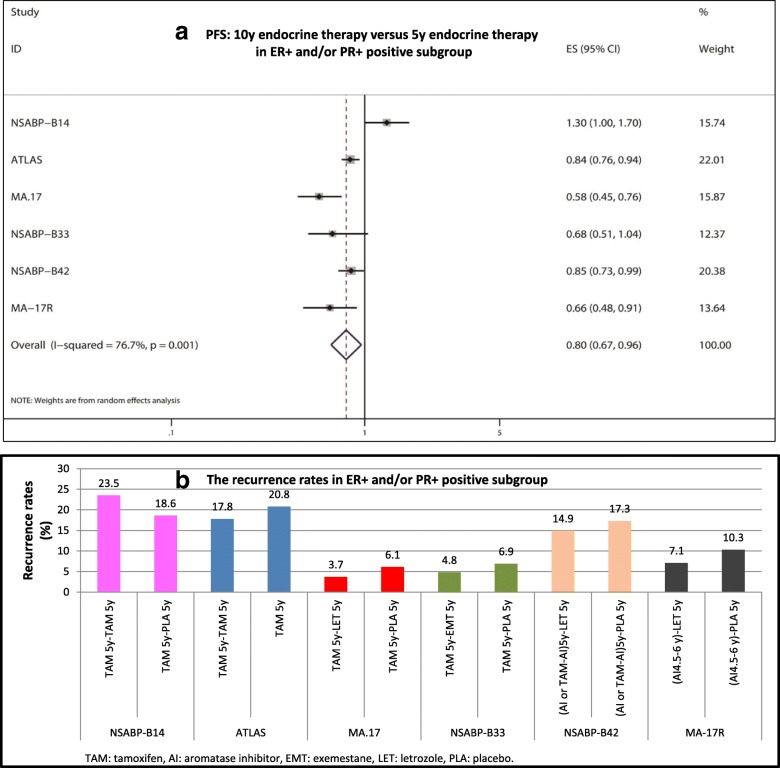
DFS analysis in ER+ and/or PR+ positive subgroup. **a** PFS: 10y endocrine therapy versus 5y endocrine therapy in ER+ and/or PR+ positive subgroup, **b** The recurrence rates in ER+ and/or PR+ positive subgroup

As shown in Fig. 6a and b, six trials reported the data of HR/RR and 95% CIs for DFS in the ER+ subgroup and/or PR+ subgroup. There was significant heterogeneity among the studies; thus, REM analysis was conducted (I^2^ = 76.7%, *P* = 0.001). ER+ and/or PR+ subgroup analysis showed that extended endocrine therapy to 10 years significantly improved DFS compared with 5 years of endocrine therapy (HR = 0.80, 95% CI: 0.67–0.96). In the NSABP-B14 trial, the recurrence rate in the TAM 5y - TAM 5y group was higher than that of the TAM 5y group (23.5% versus 18.6%, respectively). However, in the ATLAS, MA.17, NSABP-B33, NSABP-B42, and MA-17R trials, the recurrence rates in the extended treatment groups were lower than those in the 5 years of endocrine treatment group (17.8% versus 20.8%, 3.7% versus 6.1%, 4.8% versus 6.9%, 14.9% versus 17.3%, and 7.1% versus 10.3%, respectively).

## Discussion

Five years of adjuvant endocrine therapy has been verified to significantly reduce the recurrence risk and cancer mortality in ER+, early-stage breast cancer [20]. Whether extended endocrine therapies could further increase the clinical benefit has always been a controversial topic. Some trials have shown that extended endocrine therapies could further lower the recurrence risk. However, IDEAL trials demonstrated that (AI/TAM/TAM-AI) 5y - LET 5y did not bring significant prolongation for DFS and OS compared with (AI/TAM/TAM-AI) 5y - LET 2.5y [8]. Additionally, in the Scottish and NSABP-B14 trials, extended TAM to 10 years was confirmed to decrease DFS compared with 5 years of treatment [12, 13]. The optimal time of extended endocrine treatment is a controversial hot topic for oncologists. Our study comprehensively analyzed the related clinical trials, and the results showed that extended 10 years of adjuvant endocrine treatment was more efficacious than “standard” 5 years of endocrine treatment in preventing recurrence. Unfortunately, extended endocrine therapy to 10 years are not verified to improve OS, and this might mainly influenced by the efficacy of multiline therapy after relapse. It should not be overlooked that the overall follow-up time is not long, so a very long term follow-up is needed in these populations to prove OS differences. Considering the difference in the research populations and backgrounds in different trials, clinical application of extended endocrine treatment should be carefully weighed.

The debates concerning extended TAM treatment is obvious. The time of extended endocrine therapy is an important factor affecting the conclusion. The ATLAS and aTTom trials reported that extended adjuvant TAM for more than 10 years provides further protection against recurrence; however, 5–9 years of application did not exert a positive effect [9, 10]. Saphner reported that the mean recurrence rate of 5–10 years was 4.3% per year; 5–9 years of extended endocrine treatment might not be the most advantageous with a lower recurrence risk [21]. With the increase in the recurrence risk after 10 years, extended adjuvant TAM for more than 10 years probably shows significant clinical efficacy. In the process, identifying patients with high-risk recurrence is the key factor to guide treatment.

The difference in drugs in extended endocrine treatment might affect the conclusion. Coombes RC reported that the DFS and OS in the (TAM-EXE) 5y group were significant improved compared with those in the TAM 5y group [22]. The 10-year breast cancer mortality was 12.1% after 5 years of AI treatment, a value that was lower than 14.2% after 5 years of TAM treatment [23], and 5 years of AI showed an advantage over 5 years of TAM in decreasing the 10-year mortality risk. The extended 3 years of ANA after 5 years of TAM showed a significant DFS reduction of 36% (ABCSG-6a) [24], and the extended 5 years of LET after 5 years of TAM showed a significant DFS reduction of 42% [3]. Our subgroup analysis showed that TAM 5y - AI 5y and (AI and/or TAM) 5y - LET 5y significantly prolonged DFS compared with TAM 5y. These data indicated that extended AI treatment is a valid therapeutic option for early breast cancer. However, the conclusions of continuous 10 years of TAM were not consistent. The NSABP-B14, Scottish and E4181/E5181 trials reported that an additional benefit was not observed in the TAM 5y - TAM 5y arms [11–13]. However, the ATLAS and aTTom trials reported that a prolonged 10 years of TAM significantly reduced the recurrence risk [9, 10]. Our subgroup analysis verified that continuous 10 years of TAM did not improve DFS compared with 5 years of TAM.

The following factors are closely related to the efficacy of extended endocrine therapy: (1) Hormone receptor status. In hormone receptor-positive patients, 5 years of TAM treatment decreased the 5-y and 10-y breast cancer recurrence rates from 26.1 and 37.7% to 15.4% and 24.8%, respectively; however, the treatment could not decrease the recurrence rates in hormone receptor-negative patients [25]. In 6 ER+ and/or PR+ trials, five trials (ATLAS, MA.17, NSABP-B33, NSABP-B42, MA-17R) showed the superiority of 10 years of endocrine treatment [4–7, 9], whereas only the NSABP-B14 trial reported an opposite conclusion [13]. Our comprehensive analysis showed that 10 years of endocrine treatment further reduced the breast cancer recurrence rate and significantly improved DFS compared with 5 years of endocrine treatment (HR = 0.80, 95% CI: 0.67–0.96) in ER+ and/or PR+ patients. (2) Menstrual status. The ATLAS stratified study showed that extended TAM treatment seemed to have a beneficial effect in preventing recurrence in postmenopausal women (RR = 0.85, *P* = 0.05) but has no effect in premenopausal women (RR = 0.81, *P* = 0.15) [9]. In postmenopausal women, 5y TAM - 5y AI led to significant improvement in DFS; in the MA.17 trial, it led to OS improvement in the high-risk node-positive subset [4]. Our meta-analysis showed that extended endocrine therapy to 10 years significantly improved DFS in postmenopausal women (HR = 0.70, 95% CI: 0.58–0.85). (3) Lymph node status. The late recurrence risk in breast cancer patients with more than three positive nodes was 2.18-fold that in patients without lymph node metastasis after the completion of 5 years of adjuvant endocrine therapy [26]. In theory, the extended endocrine therapy should exert a clinical benefit in these patients with a lymph node-positive status. Unfortunately, the ATLAS trial showed that 10-year TAM treatment could not improve DFS compared 5 years of TAM in lymph node-positive or -negative patients [9]. 5y TAM-5y AI treatment only improved DFS compared with 5y TAM treatment in lymph node-positive patients, and the corresponding results in lymph node-negative patients were uniform [4, 7]. The mode of TAM 5y -AI 5y is worth recommending in lymph node-positive patients.

The clinical efficacy and side effects are two determining factors for extended endocrine treatment. Serious adverse effects will interrupt the persistence of endocrine treatment [27, 28]. The Ideal study reported that 15.7% patients discontinued extended 2.5 years of LET treatment because of toxicities [8]. However, the MA17R trial showed that it was safe and beneficial for women with HR-positive breast cancer to receive AI for another 5 years after initial treatment; the incidence of toxic effects was lower except that bone-related events occurred more frequently than those in the placebo group (14.0% versus 9.0%, respectively) [6]. Moreover, the discontinuation rate of extended AI for 5 years was 6.0% due to bone fracture, a value similar to that for the placebo. Extended AI to 10 years may be recommended when improved DFS is accompanied by a lower incidence rate of toxic effects. The reported ratios of endometrial cancer in the TAM 5y - TAM 5y group were 2.1, 1.7 and 2.2 folds compared with those in the TAM 5y group in the NSABP-B-14, ATLAS and aTTOM trials, respectively [9, 10, 13]. The TMA 5y - TAM 5y strategy may not be recommended with no improvement in DFS and higher incidence rates of endometrial cancer. Sequential treatment with different types of endocrine drugs is also used to maintain the efficacy to a maximum and decrease adverse effects to a minimum.

## Conclusion

Based on standard 5-year endocrine treatment, extended endocrine treatment to 10 years could further bring a DFS benefit for patients with early breast cancer, especially in the AI and/or TAM 5y - AI 5y mode, ER+ subgroup and/or PR+ subgroup, postmenopausal subgroup and lymph node-positive subgroup. Of course, the recognition of patients with the highest recurrence risk will help to obtain more clinical benefit from extended endocrine treatment, and gene analysis or molecular markers will be used to guide individualized endocrine therapy.

## Additional files

Additional file 1: **Table S1.** PRISMA Checklist. (DOC 67 kb)

Additional file 2: **Figure S1.** DFS subanalysis of 10-y endocrine therapy versus 5-y endocrine therapy. (PDF 191 kb)

Additional file 3: **Figure S2.** DFS and OS analysis of > 8 years of endocrine therapy versus < 8 years of endocrine therapy. (PDF 184 kb)

## Abbreviations

AI: Aromatase inhibitor
DFS: Disease-free survival
EMT: Exemestane
FEM: Fixed-effects model
LET: Letrozole
NSABP: National Surgical Adjuvant Breast and Bowel Project
OS: Overall survival
REM: Random-effects model
TAM: Tamoxifen
