# Supplementary figures and images for: Clinical outcomes comparison of 10 years versus 5 years of adjuvant endocrine therapy in patients with early breast cancer
Source: BMC Cancer. 2018 Oct 12;18:977. doi: 10.1186/s12885-018-4878-4 (PMC6186070; doi:10.1186/s12885-018-4878-4)

Figure S1

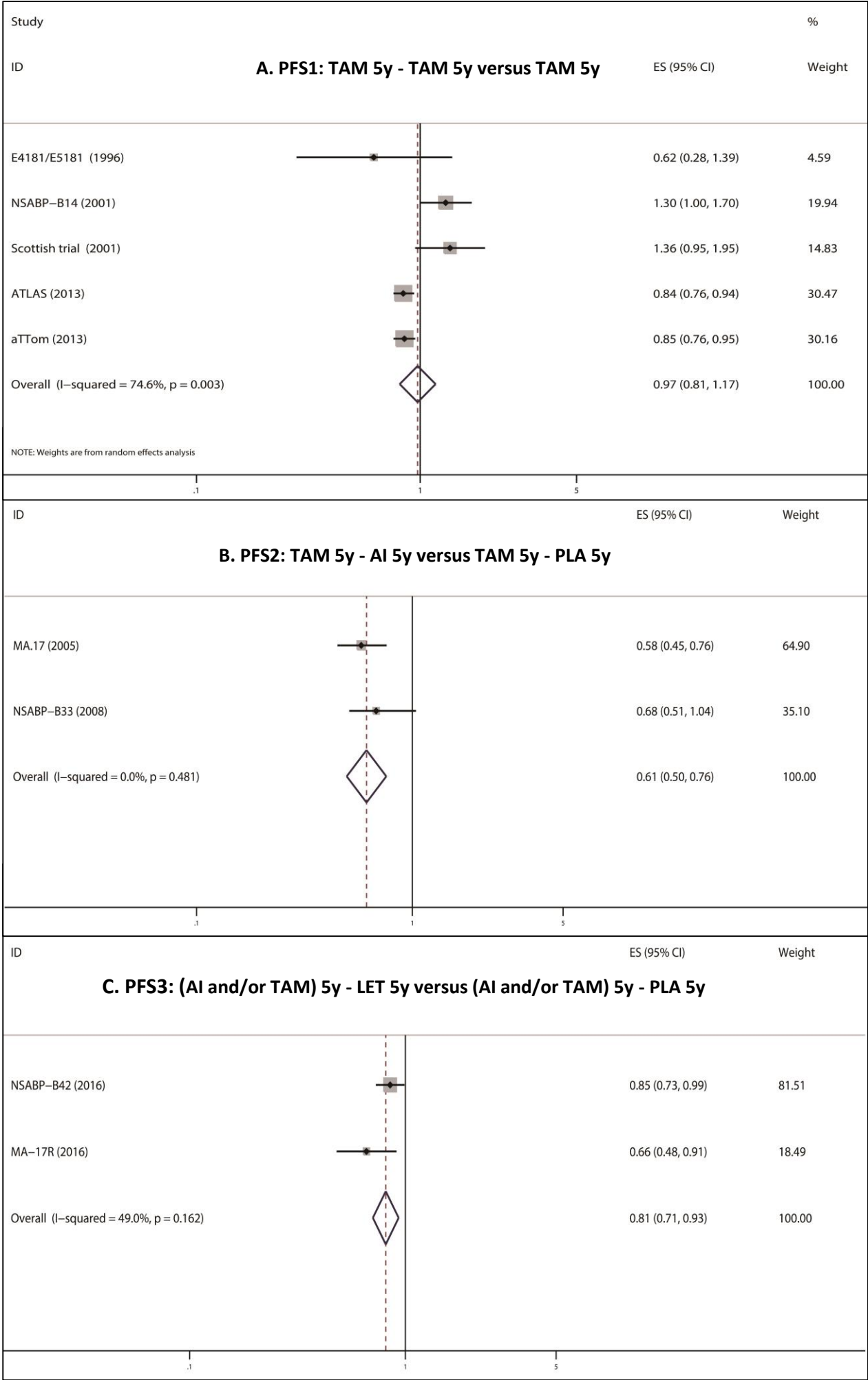

Supplement: Supplementary file 2 — Figure S1. DFS subanalysis of 10-y endocrine therapy versus 5-y endocrine therapy. (PDF 191 kb) [file 12885_2018_4878_MOESM2_ESM.pdf]

Figure S2

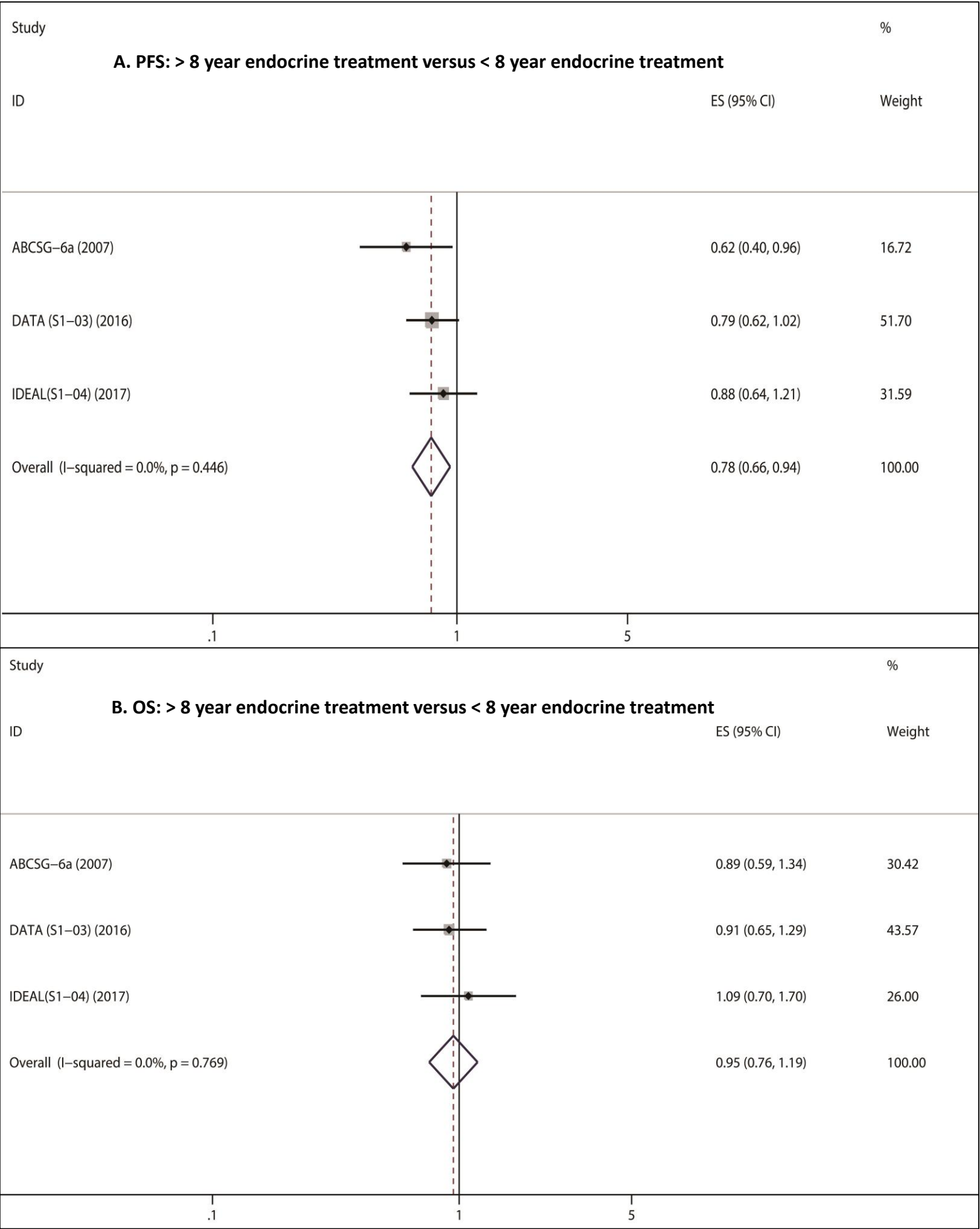

Supplement: Supplementary file 3 — Figure S2. DFS and OS analysis of > 8 years of endocrine therapy versus < 8 years of endocrine therapy. (PDF 184 kb) [file 12885_2018_4878_MOESM3_ESM.pdf]
